# A Qualitative Study on the Implementation of Flexible Assertive Community Treatment – an Integrated Community-based Treatment Model for Patients with Severe Mental Illness

**DOI:** 10.5334/ijic.5540

**Published:** 2021-04-29

**Authors:** Camilla Munch Nielsen, Carsten Hjorthøj, Merete Nordentoft, Ulla Christensen

**Affiliations:** 1 Copenhagen Research Center for Mental Health (CORE), Mental Health Center Copenhagen, Denmark; 2 Copenhagen University Hospital & University of Copenhagen, Denmark; 3 Copenhagen University Hospital, Denmark; 4 University of Copenhagen, Denmark

**Keywords:** integrated care, severe mental illness, implementation, context, clinical experience

## Abstract

**Introduction:**

Flexible Assertive Community Treatment (FACT) is a model of integrated care aiming to increase continuity of care for individuals with severe mental illness. FACT teams have been implemented in many countries by merging Assertive Community Treatment (ACT) and standard care from Community Mental Health teams (CMHTs). However, there has been little research on how practitioners experienced the fusion of these teams.

**Aim:**

This study explores how former ACT and CMHT practitioners perceived the implementation of FACT.

**Method:**

Perceptions of the FACT model, implementation strategies and adaptations of the model were investigated through 17 semi-structured interviews with FACT practitioners.

**Results:**

Perceived positive contributions of FACT included the recognition that FACT addressed a service gap for a group of patients who could benefit from increased support and improved integration of outpatient and hospital care. Perceived disadvantages of FACT included the experience that FACT drew away resources from ACT patients with the highest psychiatric needs. The findings also describe barriers to implementation, such as lack of perceived benefit of FACT, the maintenance of traditional work culture and insufficient organisational capacity. These challenges required the negotiation of local implementation strategies and adaptations.

**Conclusion:**

FACT increases access to intensive care for a broader group of patients with severe mental illness. However, findings from this study also suggest that the increased caseload in FACT compared with ACT and a changed mindset may not reflect the needs of the smaller group of patients who find it difficult to engage with mental health care services.

## Introduction

Initiatives to improve continuity of care tops the mental healthcare agenda in many Western countries [1,2]. Integrated care for individuals with severe mental illness is assumed particularly important as their needs span across primary, secondary and social care [3]. Since the 1960s there has been a move toward the reduction of psychiatric hospital beds in favour of treatment outside the hospital [4]. Various models of integrated community mental health care have been developed including Assertive Community Treatment (ACT) and Flexible Assertive Community Treatment (FACT) [4,5,6].

The ACT and FACT model share common goals such as aiming to meet a wide range of patient needs rather than arranging for help to be provided by external services [5,6,7]. This includes illness management, rehabilitation, housing, and finances. A key feature of both models is to provide a high amount of outreach contacts (visiting the patient at home or other local surroundings) in contrast to office-based appointments. Outreach contacts also involve visiting patients who are admitted to inpatient care. Furthermore, both models of care use a team-based approach with shared caseload. During shared caseload team members share responsibility for their patients and several team members work together with the same patient [6,8].

One of the main differences between the ACT and FACT model is the target group (supporting ***Table 1***). ACT is an evidence-based model specialized in providing intensive treatment to patients with the most severe mental illness [5]. These patients often have difficulties engaging with standard treatment and have a high use of psychiatric services. Assertive outreach is a central feature of ACT which involves persistent attempts to contact patients to make or maintain engagement with services. To achieve this, the ACT team operates with low caseloads [9]. In many countries, patients are transferred from ACT teams to Community Mental Health teams (CMHTs) when they no longer need intensive services. CMHTs primarily provide treatment by individual case management. The key aspect of individual case management is that each practitioner work independently with a group of patients. CMHTs have higher caseloads than ACT, so contacts with the patient are primarily offered at the team office, and more services are provided by external providers [9].

**Table 1:** Major themes organised within a frame of implementation theory.


**Theme 1: Reactions to the organisational change** This theme concerns the beliefs and attitudes of FACT held by the interviewed practitioners, which involves perceptions related to the need for and potential benefits of FACT.^a^

**Theme 2: The integration of two organisational cultures** This theme describes the integration of ACT and CMHT to establish FACT and the organisational cultures underlying the practitioners’ behaviour.^b^

**Theme 3: FACT model characteristics – implementation strategies and challenges** This theme refers to characteristics of the FACT model and how applicable they were with daily practice, as seen from the perspective of the practitioners. We also identified adaptation processes to fit the practitioners’ preferences or organisational capacity.^c^


^a^ Based on the concept “mental models” described by Nielsen and Randall. ^b^ Based on the concept “organisational culture” described by Nielsen and Randall. ^c^ Based the concept “model characteristics” and “adaptations” described by Durlak and Dupré.

The FACT model provides treatment to a broader group of patients by blending elements of ACT and individual case management [6]. The flexibility of the FACT model is the ability to upgrade and downgrade the level of care within the same team. When intensive treatment is needed, the patients are supported by a team-based approach with shared caseloads. Names of patients in need of intensive FACT services are listed on an electronic board and these patients are discussed at daily team meetings where it is decided which team members will visit the patient. The care is downgraded to less intensive individual case management when the patient has stabilised [6]. At both levels of care, the FACT team provides a high amount of outreach contacts [6]. Finally, a feature of the FACT model that differs from ACT is the employment of a peer worker in the teams to support the focus on rehabilitation.

FACT was developed in the Netherlands and has disseminated rapidly to many European countries despite limited evidence for its effectiveness [10,11,12,13,14]. In Denmark, FACT teams have been implemented by merging ACT teams and CMHTs. The management board for Mental Health Services in the Capital Region of Denmark decided to implement FACT to address challenges in transitions of patients after a period of stability from ACT to CMHT [15]. They expected to improve the continuity of care by merging these teams into FACT teams because the FACT model allows the intensity of care to be adjusted within the same team. However, due to the scarce evidence base for the effectiveness of FACT, it was decided to test and evaluate FACT before wide implementation. The effect of FACT was evaluated in a large clinical controlled study comparing treatment from FACT teams with treatment from CMHTs and ACT teams [15]. Results of the clinical study showed that there was no difference in the number of inpatient days between patients receiving FACT and patients receiving ACT or CMHT. However, FACT teams provided a more intensive service in terms of increased outpatient contacts.

This qualitative study is embedded in the effect evaluation of FACT in Denmark to provide knowledge about experiences with the implementation of FACT teams in Denmark. Qualitative studies nested in clinical studies of complex interventions can be used to provide information about the context in which the model is delivered [16]. The implementation of the FACT model may vary between the Netherlands and Denmark, as an intervention with many components often makes it difficult to standardise the delivery in different contexts [17]. Relating effects of the model to the context can offer valuable information about how the model works and why impacts may vary from one setting to another [16]. One Swedish study showed that FACT was well received by clinicians, which may be a significant driver of the rapid implementation of FACT [18]. FACT was implemented in Sweden by replacing CMHT; however, in Denmark and other countries, FACT was established by merging ACT and CMHTs (9,17). To date, there is little knowledge about the clinical experiences of this fusion in the establishment of FACT, and the only study that has examined the implementation of FACT has focused on identifying contextual factors contributing to rapid dissemination and implementation with high fidelity [19]. Thus, research on the strategies and adaptations facilitating the implementation of the FACT model characteristics is lacking.

### Aim

To develop an understanding of how mental health practitioners experienced FACT and its actual use in daily practice and what strategies or adaptations were chosen to make FACT more suitable for a Danish mental health care context.

### Implementation context

The focus of this study is on the implementation of the first five Danish FACT teams that were established in an urban and a rural mental health care centre in the Capital Region of Denmark in May 2016. Fidelity assessments were conducted in all five FACT teams 9 months after implementation using the FACT fidelity scale [20]. Four out of the five teams was assessed as operating with good fidelity while the last team had insufficient fidelity [15]. The teams’ caseload ranged from 20–30 patients per case manager. The urban FACT teams had higher caseloads than the rural teams, but the travel distances were shorter, and they could often travel by electronic bikes to visit the patients. The rural FACT teams had to travel longer distances by car to provide outreach care. One peer worker was employed in each of the FACT teams. The rest of the staffing was based on the existing group of employees from ACT teams and CMHT. Two project leaders employed by the Mental Health Services in the Capital Region of Denmark supported model implementation including training, consultation and administrative support. All employees received several days of training to provide knowledge about the rationale behind FACT, engage and support the new teams in getting started and offer information about the FACT manual and the model characteristics. A steering group was assembled with managers and employees from the urban and rural FACT setting and the two project leaders. The steering group met regularly to align and share experiences with model implementation. Furthermore, a group of managers and employees visited FACT teams in the Netherlands to gain inspiration and knowledge from the original Dutch FACT teams. At the time of this study, the Danish FACT programme contained key elements of the FACT model including daily FACT board meetings using a shared caseload approach, outreach care, activities aiming at integrating FACT and inpatient care, and the employment of a peer worker. Other elements of the FACT model that were implemented later included the integration of vocational assistance in the teams and the establishment of stronger collaboration with community services.

## Theory and Methods

### Theoretical framework

Development of the interview guide and analysis of interview transcripts were organised and interpreted within a frame of implementation theory to gain an understanding of FACT implementation in the Danish context. We will not cover all dimensions of context [16], but instead focus on the organisational context, primarily the perceptions of the providers and the organisational culture described by Nielsen and Randall [21] as well as intervention characteristics and adaptations conceptualised by Durlak & Dupré [22]. According to Nielsen and Randall, successful implementation often depends on changing behaviour among the providers and thus it is crucial to understand their mental models i.e. underlying attitudes and values [21]. The extent to which providers perceive a need and benefit of the intervention influences their motivation to engage in activities and may explain how the intervention is received, modified or resisted. If change is forced and providers feel excluded in decisions about change, they may be less engaged in the implementation process. The organisational culture refers to how well the intervention fits with the current work culture of the staff and existing practices [21]. Furthermore, according to Durlak and Dupré, it is essential to assess how compatible model characteristics are with the needs and priorities of the organisation, and the degree to which these characteristics can be adapted [22]. Adaptation occurs when model characteristics are adjusted to match the needs of the providers or organisational capacity. In a review study of previous research on implementation, Durlak and Dupré argue that adaptations should not only be seen as a failure to achieve fidelity but also as a way to enhance acceptability and applicability of the intervention [22].

### Methods

This qualitative study is based on interviews with former ACT and CMHT practitioners from three urban and two rural FACT teams in the Capital Region of Denmark. We conducted a total of seventeen semi-structured interviews, (nine interviews with former CMHT practitioners and eight interviews with former ACT practitioners). We sampled participants purposively to include FACT practitioners with prior work experience in either ACT or CMHT and to represent all disciplines in FACT including nurses, psychiatrists, psychologists, social workers, occupational therapists and peer workers. However, we decided to exclude interviews with peer workers because they had only been working in FACT for a short time when we conducted the interviews. Furthermore, participants were recruited from both rural and urban FACT teams to investigate how geographic factors may influence FACT implementation. We provided information about the study to the FACT team leaders, and they selected 12 participants based on profession, prior work experience and workplace setting. These interviews were conducted from June to August 2017, approximately a year after the implementation of FACT. Most of the participants turned out to have a background in CMHT because there was an imbalance of CMHT practitioners in FACT, and some ACT practitioners had left FACT. To strengthen the sample specificity [23], we decided to recruit five additional ACT practitioners in the early stage of implementation from other FACT teams (August 2017 and February 2019). These five practitioners were found through the researchers’ network. The interviews lasted on average about 60 min (range 30 to 80 min).

The interview guide included themes to elicit participant perceptions of FACT and the implementation of FACT activities, including adaptations of the model and factors that facilitated or hindered implementation (see supporting text 1). The interviews were recorded, transcribed verbatim and coded using Nvivo 11 software.

#### Analysis

The interviews were analysed using systematic text condensation, which is a four-step method of thematic cross-case analysis [24]. First, the transcripts were read in full length to get a general impression of the data, and preliminary themes associated with the research questions were identified. Second, we searched the interviews systematically for meaningful pieces of text describing perceptions of the FACT model, implementation strategies, adaptations of FACT and contextual organisational factors that may have facilitated or hampered the implementation. Third, the pieces of text were coded by organising them according to the preliminary themes. Finally, themes were adjusted and further developed by rereading the transcripts with a focus on identifying similarities and differences across participants. Findings were discussed both among the authors and by an interdisciplinary team with and without clinical experience. The team included researchers in the field of clinical psychiatry, public health, and social inequality in rehabilitation and treatment. One of the authors had been involved in the previous roll-out of the Danish ACT teams. The different backgrounds of the researchers enhanced the reflexivity and interpretation of the findings [25]. To increase credibility, the interview participants had the opportunity to comment on the interpretation of their citations. They all agreed with the authors’ interpretation, so no changes were made.

### Ethics and consent

The Danish Data Protection Agency has approved the project (RHP- 2017-006). The purpose of the study was explained to all participants, and that participation was voluntary. Written informed consent for participation was obtained. The participants were anonymised and were assured of the confidentiality of the information they provided

## Results

This study showed that practitioners held different perceptions of FACT which required the negotiation of new work practices. The findings suggest that there were differences in how former ACT and CMHT practitioners perceived FACT. Therefore, all citations are specified with ACT or CMHT to link their relation to their previous work background. In the following sections, we present the three overall themes: 1) Reactions to the organisational change, 2) The integration of two organisational cultures, and 3) FACT model characteristics - implementation strategies and challenges. These themes have been developed using implementation theory as displayed in ***Table 1***.

### Reactions to the organisational change

Most of the former CMHT practitioners perceived FACT as a more efficient use of resources because a broader group of patients gained access to intensive care. They emphasised that FACT addressed a service gap for a group of CMHT patients, who could benefit from increased support in periods with destabilisation. CMHT did not have the resources to increase the intensity of care, as stated by one practitioner:

“An advantage of FACT is the ability to provide better treatment for a broader group of patients when needed. We had a group of patients in CMHT, who needed more support than we could offer, and often they did not meet criteria for ACT. A lot of ACT patients also had periods with more stability, but you were worried about the transition to CMHT, because they may relapse in three months. You can say that we make a more efficient use of the resources now.” [previous CMHT practitioner]

This CMHT practitioner also touched upon an issue related to transitions between ACT and CMHT. He explained that it could be challenging to decide when the patient was ready to transition from ACT to CMHT.

ACT practitioners were generally more reluctant to implement FACT. They were concerned that FACT would draw away resources from patients with the most severe illness, exemplified by one ACT practitioner:

“I object to the dismantling of the ACT team, when you don’t know how the most vulnerable group of patients will react, and obviously one can imagine, it is the most severely ill that will pay for an advance in CMHT.” [previous ACT practitioner]

Several ACT practitioners underlined that ACT is a clinically effective model in supporting patients who are hard to engage in standard treatment. They were concerned that the higher caseload in FACT would reduce the flexibility of assertive outreach and neglect this group. Furthermore, some experienced that the decision to replace ACT was controlled from above and felt that management did not listen to their knowledge or opinions. This resulted in resistance, and some ACT practitioners started looking for new jobs:

“I have decided to find a new job. I want to have a job, where I use the skills I have attained through many years in ACT and where I can make an impact.” [previous ACT practitioner]

This practitioner did not feel that he could use his qualifications sufficiently in FACT. FACT targeted a broader group of patients with a severe mental illness and the skills he had acquired through many years’ experience of working in ACT with a small target group of patients with mostly psychotic illness were not used in the same way.

Other practitioners from previous ACT and CMHT, assumed that the resistance against FACT among primarily former ACT practitioners was mainly a result of lost privileges and professional autonomy over their work:

“The most significant challenge for them [ACT practitioners] – as I saw it – it was, that they lost an immense privilege. I must say, you have outstanding opportunities to do a good job when having a caseload of 10 patients.” [previous CMHT practitioner]

These findings suggest that there were considerable differences in how practitioners related to FACT and whether the changes aligned with their professional values and job motivation.

### The integration of two organisational cultures

Several practitioners stated that there was a need to discuss the implementation of the new work procedures. Team members often had different approaches depending on whether they had a background in ACT or CMHT, and this led to discussions about the amount and content of successful treatment.

One CMHT practitioner depicted that a team member from former ACT would respond to missed appointments differently than she was used to:

“I think that particular one of our previous ACT practitioners, who has a big heart for the most severely ill, approaches the patient in a different way than I do. If a patient misses an appointment, I make a telephone call or write a text message to make a new appointment. I won’t go to the patient’s home and look for them.” [previous CMHT practitioner]

As indicated above, it could especially be difficult to make decisions on a team level about patients, who had difficulties attending appointments or who needed the intensive services. Practitioners discussed discrepancies in work practice and aligned values to find a mutual understanding about the delivery of the treatment. One of the discrepancies discussed was how many inputs the FACT team could provide. Several ACT practitioners described that the ACT approach as a move beyond a focus on medication, and the ACT team had resources to offer help with practical matters, finances or engage in social activities with the patient. They experienced that there was less focus and time for these activities in FACT:

“It happens that you don’t always reach the patients that need things a bit different (…). Previously we could say, you can always contact us, and we can help you if you have bills that need to be paid or if you need to put together your IKEA furniture (…). Sometimes that was the starting point to make contact. We don’t have the same opportunities now.” [previous ACT practitioner]

Some practitioners from CMHT argued that it was essential to make a clear distinction between medical treatment and social care services. They stated that the nonmedical issues were a responsibility for social services and encouraged a stronger collaboration with this sector. One practitioner specified that it was necessary to discuss the different approaches and responsibilities in the team to even out imbalances between ACT and CMHT practitioners:

“We need to discuss approaches in the team. Whether you use a socio-educational or health approach. I think that is where we clash.” [previous CMHT practitioner]

It appeared from the interviews that the integration of ACT and CMHT was an organisational change that challenged prior work routines and ways of thinking and necessitated the negotiation of new work processes to integrate these cultures.

### FACT model characteristics – implementation strategies and challenges

This theme describes how the following key characteristics of the FACT model were received and implemented: 1) FACT board meetings with the shared caseload, 2) outreach care, and 3) integration of care between FACT and inpatient services.

#### Characteristic 1: FACT board meetings and shared caseload

##### Improved teamwork and shared knowledge

**Figure 1:**
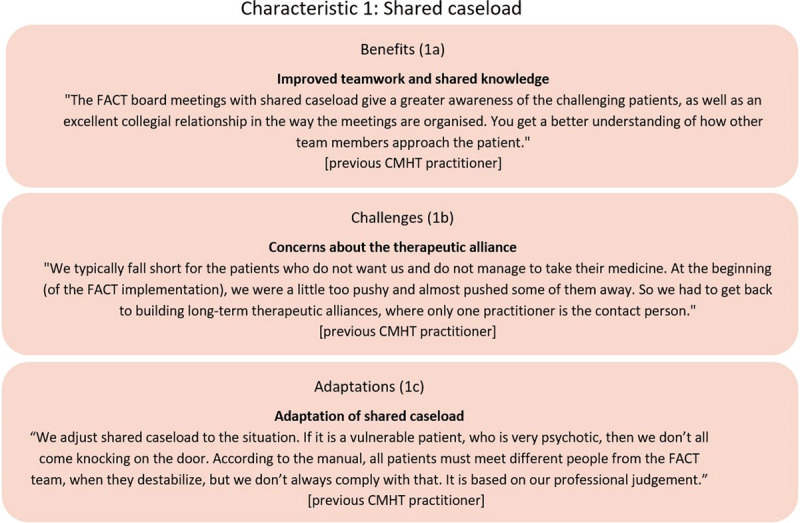
Example of FACT practitioners’ descriptions of benefits, challenges and adaptations of shared caseload.

One of the most cited benefits of FACT was the experience of improved teamwork facilitated by the daily FACT board meetings with the shared caseload (***Figure 1a***). Practitioners from both ACT and CMHT stated that the way the FACT board meetings were organised led to better communication between team members and created awareness of the patients with increased needs. They also had a better idea of how the various professions and competencies could contribute to support these patients. One practitioner also mentioned that the shared caseload procedure made it more “*natural*” to ask other team members for help:

“The daily meetings make help from other team members more accessible. You could also ask for help previously, but now it feels more legal.” [previous CMHT practitioner]

Especially former CMHT practitioners underlined that the shift from working independently to working as a team made them feel less alone and better supported when a patient destabilised.

##### Resistance towards shared caseload and adaptations of the approach

It was a concern among some practitioners that sharing the care of a patient between the team members could threaten the therapeutic alliance (***Figure 1b***). These practitioners were especially concerned about the individuals who were hard to engage in treatment:

“The patients who are very ill, who are dependent on a consistent relationship and have gained trust in one or two case managers, they will lose.” [previous ACT practitioner]

Sometimes the practitioners would modify the extent of shared caseload to adjust it to the needs of the most vulnerable patients (***Figure 1c***). In situations where the patient would only agree to have contact with their case manager, the practitioners mentioned that they supported the case manager by taking over other tasks as a strategy to help with the workload. Some ACT practitioners more directly resisted shared caseload. They expressed that shared caseload was practised differently in ACT where the case manager would team up with another team member, so at least one practitioner was familiar to the patient. A few ACT practitioners noted that they avoided writing the name of the patient on the FACT board if they believed that the patient would not benefit from contact with other team members:

“If I don’t think it makes sense to involve the other team members, then I don’t always record the name of the patient on the board during a period of destabilisation. I just give the patient more of my own time” [previous ACT practitioner]

As implied above, a group of practitioners did not always see the appropriateness of shared caseload. One practitioner explained that part of the reluctance had roots in the past. Team members often hang on to habits of working individually and had to trust that other team members could achieve good contact with the patient.

In general, the practitioners perceived the implementation of shared caseload as challenging. Still, several practitioners argued that there was a need to focus on the benefits of the approach and underlined that forming good relations with more than one team member was crucial to protect the patients against staff turnover. They observed that most patients accepted to meet other team members. Some practitioners stated that they had gradually changed their perception of shared caseload and become more motivated to share the patients with the team:

“We anticipated that patients would find that it was challenging to meet different people, but my impression is that they accept it to a much greater extent than we thought and that they often see it as a positive thing.” [previous CMHT practitioner]

#### Characteristic 2: Outreach work

##### Perceived benefits of outreach

**Figure 2:**
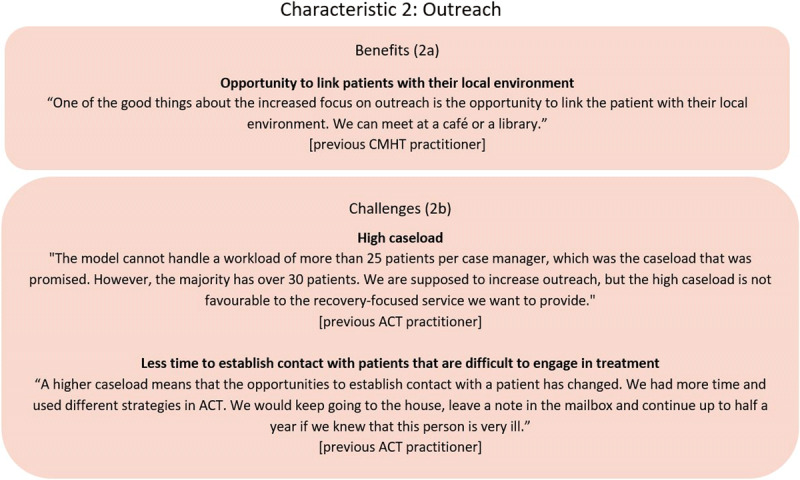
Example of FACT practitioners’ descriptions of benefits and challenges of outreach work.

Several practitioners stated that it was an advance to community care that the FACT model encouraged more of the contacts as outreach counselling – also for stable patients. They explained that outreach was an opportunity to identify what support the patient need in their everyday living and link individuals with resources in the community (***Figure 2a***).

##### Questioning the relevance of outreach for all patients

Other practitioners were sceptical about outreach for the more stable patients. They questioned whether outreach is necessary if the patients are not reluctant to keep appointments at the clinic.

“Sometimes there is a little too much” home is best. “For some patients, it is good to practice keeping an appointment at the FACT facility and receive the treatment services we have here.” [previous ACT practitioner]

##### Implementation challenge: high caseload and long travel distances

For the urban FACT team, a high flow of patients was one of the contextual barriers to outreach. Some practitioners warned that the rising caseload exceeded the recommended caseload in the FACT manual and threatened the flexibility of FACT to maintain regular and frequent outreach care (***Figure 2b***). Furthermore, some ACT practitioners described that the higher caseload in FACT was a barrier in efforts towards engaging the group of patients who did not respond attempts to establish contact or who declined services. They experienced that the FACT team would more easily give up on these patients (***Figure 2b***). In the rural FACT teams, a barrier to outreach was long travel distances. However, despite travel distance, the rural FACT teams aimed to provide a high amount of outreach contacts. To overcome the difficulties of travel time they tried to visit a group of patients in the same area. During the daily FACT morning meetings, each practitioner informed about their route and team members could switch appointments, so they met with patients in the same area.

##### Implementation challenges: The role of the psychologist and social worker in outreach work

Nurses and occupational therapists were appointed case managers in the Danish FACT team. The other professions in the team did not have a caseload, but complemented the work of the case manager. There were diverging opinions about the role of these “specialist disciplines” in outreach work. With only one psychologist and social worker in each FACT team, the practitioners questioned whether the team should use their resources for outreach work. Especially when these professions did not manage medication:

“We have only one psychologist and social worker in the team, so they are a limited resource. Moreover, they don’t manage medication. If they make a home visit and the patient needs medicine, then we need to send a nurse anyway.” [previous CMHT practitioner]

#### Characteristic 3: Integration of care between FACT and inpatient services

##### Establishment of common goals and better follow-up after an admission

**Figure 3:**
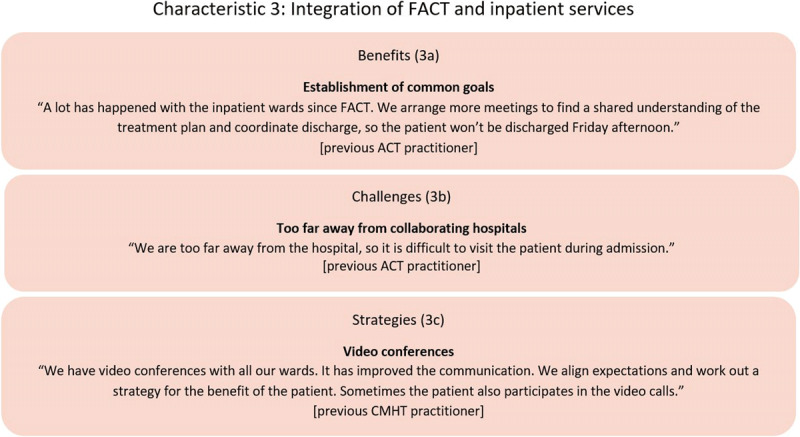
Example of FACT practitioners’ descriptions of perceived benefits, challenges and strategies regarding the integration of FACT and inpatient services.

Most practitioners mentioned that the FACT teams focused on a strong collaboration with inpatient clinics to create a shared vision about treatment plans and arrangements for discharge. This meant that the FACT team could provide a closer follow-up after discharge and arrange support for the patient when transferring from the hospital (***Figure 3a***).

##### Implementation challenges: contact with patients during admission and the collaboration with several inpatient wards

Visiting the patients during a hospital stay was mentioned as challenging due to time constraints and geographical distance (***Figure 3b***). Especially in the rural FACT team, the travel distances were often too long to visit the patient during an admission and participate in meetings with the inpatient staff. In the urban FACT teams, the shorter distances to the hospital gave them better opportunities for visiting the patients and the practitioners would often visit several patients from the FACT team to make more use of their time.

Another challenge underlined by FACT practitioners in both the rural and urban setting was the collaboration with several inpatient wards and hospitals:

“We collaborate with four inpatient wards, so the communication is widely spread. There are too many hands.” [previous CMHT practitioner]

One practitioner mentioned that the ideal scenario would be an inpatient ward with designated FACT beds located at the same hospital, so the FACT team could create a stronger collaboration with the inpatient staff.

##### Implementation strategy to integrate outpatient and inpatient care

The rural FACT teams used video calls to overcome the barrier of collaborating with hospitals far away. They discussed the treatment plan and discharge. Sometimes the patient would also participate in the video calls (***Figure 3c***). One of the FACT teams also invented a “roadshow”, where they gave presentations at all collaborating inpatient clinics to share knowledge of how FACT aims to integrate services with the inpatient clinic and create a better understanding of the different work cultures:

“We made a roadshow for all the inpatient wards. The roadshow was a presentation of FACT and transmural care. We wanted to get familiar with the staff and reduce barriers between our cultures.” [previous CMHT practitioner]

## Discussion

This study showed how variations in work culture between ACT and CMHTs highly shaped the practitioners’ perceptions of FACT. Former CMHT practitioners were more likely to emphasise positive perceptions of FACT. They depicted that the daily FACT meetings with shared caseload contributed to a better quality of care by supporting the patient with inputs from various team members, protected the patients against staff turnover and gave the team a better overview of the patients in need of intensive support. Furthermore, they accentuated that the team approach improved the work environment by making help from other team members more accessible. These perceptions shaped their motivation for implementing change and highly mirrors the findings from a Swedish study on the practitioners’ perceptions of FACT [18]. Furthermore, CMHT practitioners echoed concerns raised in other studies regarding the fragmentation between ACT and CMHTs, which may have hindered the delivery of appropriate levels of support and continuity of care [7,26]. They perceived that the integration of these services in FACT to serve a broader population of patients in periods with destabilisation was a better distribution of resources.

At the other end of the scale, some practitioners, mainly from former ACT teams, found that parts of the planned change did not make sense. ACT practitioners recognised the need for a reorganisation of community care to support CMHT patients in times of destabilisation. However, they were concerned that resources would be drained from the group of patients with the most severe illness. Former ACT practitioners emphasised the uniqueness of the ACT approach in engaging patients who are hard to reach and depicted that this approach vanished in FACT due to a changed mindset and increased caseload. To our knowledge, similar findings have not been reported elsewhere, perhaps because no other studies have investigated perceptions of practitioners in ACT teams that developed into full-scale FACT.

### Reflections on the implementation of FACT in a Danish mental health care context

The fundamental role of context in shaping how interventions work is essential when importing a new treatment model from another country [16]. Our findings highlight the importance of understanding the pre-organisational context when integrating two work cultures, as this may describe the meaning that different practitioners drew from the implementation activities [21]. We found differences in work culture between ACT and CMHTs in terms of services delivered and approaches to patient engagement, which drew parallels with other studies [27,28,29]. Implementation is also influenced by the extent to which providers see a benefit of the intervention and resistance may be found if activities do not align with their values [21,30]. Shared caseload was perceived as one of the most challenging implementation activities in this study. A team approach with shared caseload is prescribed in both the ACT and FACT model. However, some former ACT practitioners wanted to modify the degree of shared caseload because they perceived that a full team approach could be a threat to the therapeutic relationship. Shared caseload in its purest form means that the patient does not have an individual case manager and the team shares responsibility for the delivery of the treatment [5,6]. Several former ACT practitioners stated that some patients may find it difficult to relate to different team members (who they may never have met) in periods of instability. They were especially concerned about the subgroup of patients who find it difficult to engage with mental health care services. The different dimensions of the team approach have been highly debated in the literature and the operationalization of the approach varies across different ACT studies [4,31,32]. Similar to the findings of this study, there has been controversy regarding whether the whole team should be involved in the delivery of the treatment [32].

Adaptations of FACT were not only made to fit the perceptions of the providers but also the organisational capacity [22,33]. The practitioners especially mentioned that the travel distances and collaboration with several inpatient wards was a barrier for the integration of FACT and inpatient services and necessitated adjustments to fit the capacity. Adaptations of some kind are inevitable when transferring a model to a new organisational context [22]. We suggest that future studies on FACT accompany fidelity measures with descriptions of adaptations to assess the balance between the two and to provide guidance on how the model can be modified to different organisational contexts.

### Integrated care for individuals with the most severe mental illness

Three effect studies have raised concerns about whether FACT can provide intensive integrated care for individuals with the most severe mental illness [10,12,15]. The first study from the UK showed that patients who transferred from ACT to FACT had fewer admissions, bed days and outpatient contacts offset by an increase in missed face-to-face contacts. A second study from the Netherlands found significant improvements in treatment compliance, unmet needs, and quality of life for patients transferring from intensive case management teams to FACT [10]. However, a limitation of these two studies is the uncontrolled study design. The results may therefore be explained by regression to the mean resulting in an overestimation of the positive effects of FACT. A third Danish controlled study compared patients who transferred from ACT to FACT with patients in ACT control groups [15]. This study found no advantage of FACT in terms of fewer inpatient days. However, FACT patients received more outpatient contacts than patients in the ACT control groups.

This qualitative study can complement the effect studies of FACT by drawing attention to experiences with the implementation of FACT within a Danish mental health care setting. Findings from our study indicate that a smaller group of patients, with the most severe illness, may need further attention within the FACT model. Several former ACT practitioners depicted that the approaches to staying in contact with patients who had difficulties engaging with treatment were different in FACT. Former ACT practitioners described that the flexible approach of ACT to provide help with practical issues or other priorities of the patient was often the starting point to engage patients. These experiences indicate that the content of the contacts in the Danish FACT teams may be different from ACT. Additional resources and engagement techniques in the ACT model may have offered better opportunities to focus on engagement with services [9,34]. Furthermore, several practitioners depicted that they had to find a balance on how many services the team could provide and increase referrals to social services. This could be a source of discontinuity and pose a challenge, especially for the most vulnerable patients, who often find it challenging to navigate between various service providers [31].

### Strengths and limitations

This study presents novel findings of FACT implementation processes using implementation theory by Durlak & Dupré and Nielsen & Randall [21,22]. However, it is limited in its generalizability due to the local service context in Denmark. Fidelity assessments were conducted in teams that had been working with the FACT model for over 9 months. A strength of the study was that four out of five FACT teams had reached good fidelity. However, the caseload in the Danish FACT teams was higher than recommended in the Dutch standard model. Understaffing may therefore be an implementation failure that could have influenced how the practitioners’ experienced FACT. The rather small sample size (n=17) may have reduced information power; however, purposive sampling of participants and the inclusion of five additional interviews with ACT practitioners were used to increase sample specificity [23]. Information power is an indicator of the adequacy of the information contained in the sample that is relevant for the study purpose [23]. We conducted this study in the early implementation stage, which limits the descriptions of developmental processes. Furthermore, the sparse descriptions of participants characteristics may decrease the possibility for readers to assess the transferability to other studies. The reason for this restriction, however, was to preserve the anonymity of the participants. Finally, a limitation of this study was that patients’ perspectives of FACT were not assessed.

## Conclusion

Practitioners from previous ACT and CMHT teams offered valuable insight into the positive and negative contributions of FACT in the context of mental health care in Denmark. Based on practitioner interviews from this study, FACT promotes the integration of fragmented community services by providing a model where the same team can support a broad group of individuals with severe mental illness with changing needs. However, we also identified areas in need of further attention. Some practitioners raised concerns about the group of patients with the most severe illness, who find it difficult to engage with treatment. The increased caseload and a changed mindset in terms of what inputs FACT could provide compared with ACT may not reflect the needs of this population.

## Implications for clinical practice and future studies

Based on the findings above, we propose that a smaller group of patients who are hard to reach may present a clinical challenge in FACT. We suggest that future studies investigate the loss of contact with FACT services to get a clearer understanding of how FACT engages patients that are hard to reach. Furthermore, patient outcomes and experiences of FACT should be assessed in future studies.

## Additional Files

The additional files for this article can be found as follows:

10.5334/ijic.5540.s1 Appendix 1. Characteristics of the FACT, ACT and CMHT model.

10.5334/ijic.5540.s2 Appendix 2. Interview guide.
