# Supplementary material for: A Qualitative Study on the Implementation of Flexible Assertive Community Treatment – an Integrated Community-based Treatment Model for Patients with Severe Mental Illness
Source: Int J Integr Care. 2021 Apr 29;21(2):13. doi: 10.5334/ijic.5540 (PMC8086721; doi:10.5334/ijic.5540)
Supplement: Appendix 1. — Characteristics of the FACT, ACT and CMHT model. [file ijic-21-2-5540-s1.pdf]

APPENDIX 1: CHARACTERISTICS OF THE FACT, ACT AND CMHT MODEL

Characteristics	Flexible Assertive Community Treatment	Assertive Community Treatment	Community Mental Health Treatment
Target group	All patients with severe mental illness	Patients with severe mental illness, who are high service utilizers and often find it difficult to engage with health care services	Patients with severe mental illness, who are adherent to treatment and have fewer care needs
N of patients per team	250 to 300	80 to 100	250 to 300
Individual caseload	15 to 20	10	30 to 45
Team composition	Multi-disciplinary: nurses, social workers, occupational therapist, psychiatrist, psychologist, peer support worker	Identical to FACT but without the peer support worker and occupational therapist	Identical to FACT but without the peer support worker and occupational therapist
Contact with patients	Combination of home visits and office-based appointments.	Mainly home visits or meeting the patient in the local environment.	Mainly office-based.
Frequency of team meetings	Daily meetings	Frequently (up to daily)	Weekly
Approach	Team approach for individuals who need intensive care. Individual case management when the patient is stabilized	Team approach	Individual case management
**Integration of care**	Integration of intensive and less intensive community-based mental health care<br><br>Activities aimed at integrating outpatient and inpatient services<br><br>Team rather than external providers deliver the most basic needs as far as possible	Activities aimed at integrating outpatient and inpatient services<br><br>Team rather than external providers deliver the most basic needs as far as possible	Less integration of outpatient and inpatient services<br><br>Referral to external service providers
