# Supplementary material for: A Qualitative Study on the Implementation of Flexible Assertive Community Treatment – an Integrated Community-based Treatment Model for Patients with Severe Mental Illness
Source: Int J Integr Care. 2021 Apr 29;21(2):13. doi: 10.5334/ijic.5540 (PMC8086721; doi:10.5334/ijic.5540)
Supplement: Appendix 2. — Interview guide. [file ijic-21-2-5540-s2.pdf]

APPENDIX 2: INTERVIEW GUIDE

We are interested in learning from your experience working with the FACT team and discussing some of the successes and challenges so far. We will also have a few questions about how you experience your current work in FACT compared with your previous work experience in ACT or CMHT.

Introduction

1. Could you give a short presentation of yourself? What is your educational background?

Work experience before FACT – working with the ACT or CMHT team

Let's talk a bit about the time before you started working in FACT…

2. Did you work in ACT or CMHT before you started working in FACT?

3. How would you describe a typical workday before you joined FACT?
   - What was your role?
   - How did you work with the team?
   - What was the target patient population?

4. What were the key services provided by the team?
   - Home visits or office appointments?
   - Content of the service (medication management, practical help, social support)

Working with the FACT team

5. How would you describe a typical workday in FACT?
   - What is your role?
   - How do you work with the team?
   - What is the target patient population?

6. What are the key services provided by the team?
   - Home visits or office appointments?
   - Content of the service (medication management, practical help, social support)

7. How do you experience the integration of ACT and CMHT?

Implementation of FACT program characteristics

Now, let's move on and think about how key components of FACT is working so far, and how it has been developing since it was first implemented.

8. FACT board meetings and shared caseload
    - How would you describe a FACT board meeting?
    - How does the team practice shared caseload?
      Probes: collaboration, response to acute situations, sharing of patient visits across the team, teamwork compared with ACT or CMHT
    - What are the strengths and weaknesses of shared caseload and FACT board meetings?
    - What factors in the organizational context influence the delivery?
    - What strategies have your team used to implement FACT board meetings with shared caseload?
    - How has your team adapted FACT board meetings and shared caseload from the guidelines in the FACT manual?

9. Outreach
    - How does the FACT team deliver outreach services?
      Probes: for stable and unstable patients, compared with ACT or CMHT
    - What are the strengths and weaknesses of outreach in FACT?
    - What factors in the organizational context influence the delivery?
    - What strategies have your team used to implement outreach?
    - How has your team adapted outreach from the guidelines in the FACT manual?

10. Integration of FACT and hospital services
    - How does your FACT team collaborate with inpatient services?
      Probes: treatment plans, discharge, meetings, compared with ACT or CMHT?
    - How does your team keep in contact with a patient during an admission?
    - What are the strengths and weaknesses of integration of FACT and hospital services?
    - What factors in the organizational context influence the delivery?
    - What strategies have your team used to improve the integration of FACT and hospital services?
    - How has your team adapted the approach from the guidelines in the FACT manual?

General questions about FACT implementation (how FACT is working so far)

Let’s move on to the last questions about how FACT is working so far.

11. Can you give me examples of where FACT is working well in terms of program implementation? What do you think is making it work well?
12. What is not working well in terms of program implementation? How would you see the team overcoming those barriers?
13. What are the strengths of the FACT model? Is the FACT model working well for a specific group of patients?
14. What are the weaknesses of the FACT model that may need attention in the future? Is the FACT model not working well for a specific group of patients?
15. Based on your experience, what would you recommend to another organization that are implementing FACT?

Do you have any questions for me?

Thank you very much for your participation today. We appreciate your willingness to share your experiences.
